# Loss of Lkb1 impairs Treg function and stability to aggravate graft-versus-host disease after bone marrow transplantation

**DOI:** 10.1038/s41423-019-0312-3

**Published:** 2019-10-29

**Authors:** Xiuhua Su, Qianqian Wang, Wei Guo, Xiaolei Pei, Qing Niu, Maolan Liu, Yuanyuan Liu, Song Chen, Sizhou Feng, Yi He, Donglin Yang, Rongli Zhang, Qiaoling Ma, Weihua Zhai, Aiming Pang, Jialin Wei, Yong Huang, Yuechen Luo, Mingzhe Han, Xiaoming Feng, Erlie Jiang

**Affiliations:** 1 State Key Laboratory of Experimental Hematology, National Clinical Research Center for Blood Diseases, Institute of Hematology & Blood Diseases Hospital, Chinese Academy of Medical Sciences & Peking Union Medical College, 300020 Tianjin, China; 2 Department of Hematology, the First Affiliated Hospital of Kunming Medical University, Hematology Research Center of Yunnan Province, 650000 Kunming, China

**Keywords:** allogeneic hematopoietic stem cell transplantation (allo-HSCT), acute graft-versus-host disease (aGVHD), Lkb1, Treg, Bone marrow transplantation, Allotransplantation

## Abstract

Accumulating evidence suggests that a reduction in the number of Foxp3^+^ regulatory T cells (Tregs) contributes to the pathogenesis of acute graft-versus-host disease (aGVHD), which is a major adverse complication that can occur after allogeneic hematopoietic stem cell transplantation (allo-HSCT). However, the precise features and mechanism underlying the defects in Tregs remain largely unknown. In this study, we demonstrated that Tregs were more dramatically decreased in bone marrow compared with those in peripheral blood from aGVHD patients and that bone marrow Treg defects were negatively associated with hematopoietic reconstitution. Tregs from aGVHD patients exhibited multiple defects, including the instability of Foxp3 expression, especially in response to IL-12, impaired suppressor function, decreased migratory capacity, and increased apoptosis. Transcriptional profiling revealed the downregulation of Lkb1, a previously identified critical regulator of murine Treg identity and metabolism, and murine Lkb1-regulated genes in Tregs from aGVHD patients. Foxp3 expression in human Tregs could be decreased and increased by the knockdown and overexpression of the Lkb1 gene, respectively. Furthermore, a loss-of-function assay in an aGVHD murine model confirmed that Lkb1 deficiency could impair Tregs and aggravate disease severity. These findings reveal that Lkb1 downregulation contributes to multiple defects in Tregs in human aGVHD and highlight the Lkb1-related pathways that could serve as therapeutic targets that may potentially be manipulated to mitigate aGVHD.

## Introduction

Allogeneic hematopoietic stem cell transplantation (allo-HSCT) is a lifesaving treatment used for many hematologic malignancies and bone marrow failure syndromes.^1^ Unfortunately, acute graft-versus-host disease (aGVHD) is a severe complication of this procedure that impacts more than 50% of recipients.^2^ aGVHD is initiated by the activation of donor T cells, which recognize host antigens and result in a proinflammatory cytokine storm that damages target organs (e.g., skin, liver, and the gut). Although marked improvements in immunosuppressive therapies have been achieved, aGVHD is still the primary cause of nonrecurrent death^3^ associated with allo-HSCT. The development of an effective treatment for aGVHD remains crucial.

Regulatory T cells (Tregs) are generated in the thymus and are evidenced by the expression of CD4, high levels of CD25, and the intracellular expression of the transcription factor Foxp3, which are crucial for maintaining immune homeostasis via various mechanisms.^4^ Tregs have been reported to play an important role in allo-HSCT by suppressing various T cell-associated inflammatory diseases and alleviating GVHD without weakening the graft-versus-leukemia effect.^5^ This was initially verified in several murine studies,^6^ and it has been exploited in a clinical trial with early encouraging results.^7^ Meanwhile, Treg-mediated regulation of conventional T cell responses during allogeneic transplantation is a crucial mechanism of peripheral T cell tolerance that has been associated with transplantation tolerance in animal models.^8^ However, the mechanism underlying the reduction in the proportion of Tregs in patients with aGVHD has not been fully elucidated.^9^ It is vital that Treg suppressive function is ensured and that Foxp3 is continuously expressed, which is critical for maintaining the stability of Tregs. Many factors are involved; for instance, STAT5 binds to the Foxp3 promoter region, leading to stabilization of the locus.^10^ Foxp3 is also modulated by other regulators, such as Foxo1 and Foxo3a.^11^ The proinflammatory cytokine storm that occurs during aGVHD may also render Tregs more unstable via STAT3 signaling, thereby limiting the generation of Tregs.^12^

Lkb1 (encoded by the *STK11* gene) is a serine/threonine kinase that has been shown to function as a mutated tumor suppressor in Peutz-Jeghers syndrome and gynecological and other cancers.^13–15^ Lkb1 and its primary target, adenosine monophosphate-activated protein kinase, have been reported to regulate energy metabolism, cell growth, and cell polarity.^16–18^ Our previous study, which was based on conditional knockout mouse models, confirmed that Lkb1 stabilized Foxp3 expression by preventing STAT4-mediated methylation of the conserved noncoding sequence 2 (CNS2) in the Foxp3 locus, contributing to the maintenance of the identity of the Treg lineage.^19^ Subsequent articles by other researchers also identified the critical function of Lkb1 in controlling the metabolic and functional fitness of Tregs.^20,21^

Considering the significance of Lkb1 in maintaining immune homeostasis, we explored the relationship between the Lkb1 signaling pathway and the stability of Tregs in aGVHD, which might be useful in optimizing Treg-based immunotherapies. Our results show that Tregs from aGVHD patients exhibited an exhausted phenotype that was characterized by the instability of Foxp3 expression, decreased suppressor ability, defective migration capacity, and increased apoptosis, which was accompanied by Lkb1 downregulation. We also confirmed that Lkb1 deficiency could impair Tregs and thus increase aGVHD severity and revealed that Lkb1-related pathways could serve as therapeutic targets that may potentially be manipulated to mitigate aGVHD.

## Results

### Decreased frequencies of Tregs in BM and PB from aGVHD patients

**Fig. 1:**
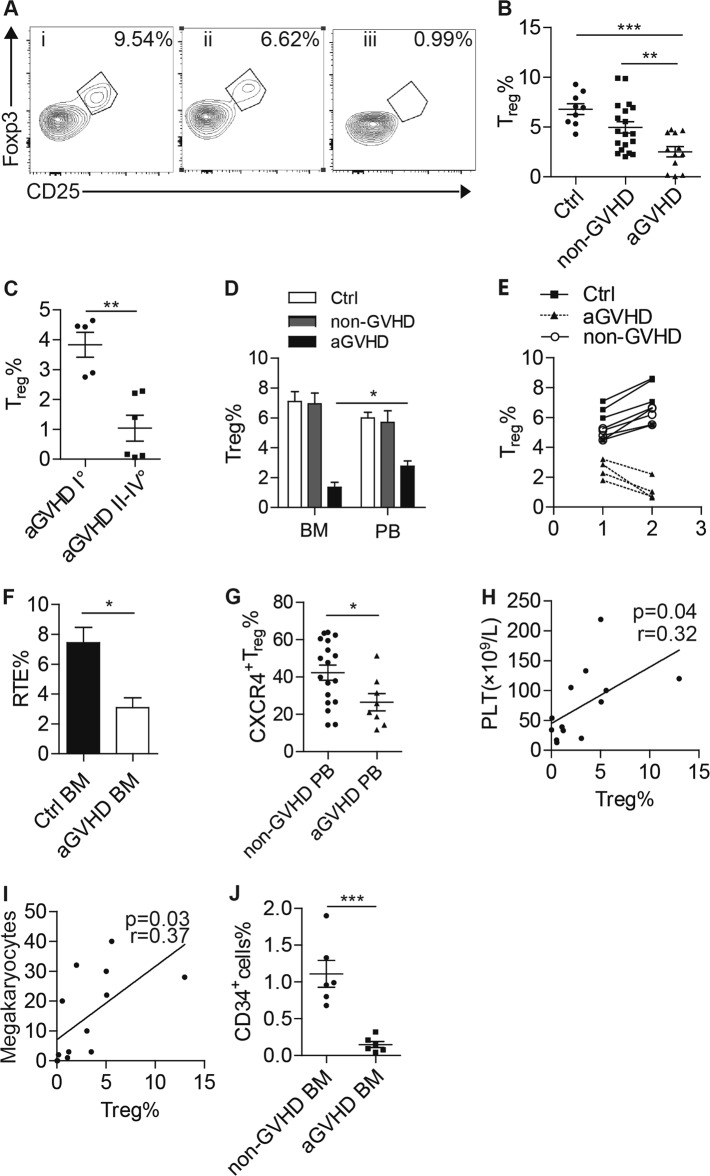
Decreased frequencies of Tregs in BM and PB from aGVHD patients. **a** Representative flow cytometric analysis of Treg frequencies in (i) healthy donor BM, (ii) non-GVHD patient BM, and (iii) aGVHD patient BM. Within the CD4^+^ T cell gate, Tregs were identified as CD25^high^ Foxp3^+^. **b** Frequencies of CD4^+^ CD25^high^ Foxp3^+^ Tregs in BM from non-GVHD (*n* = 19) and aGVHD patients (*n* = 12) and healthy controls (*n* = 9). **c** The percentages of Tregs in the BM of aGVHD I° (n = 5) and aGVHD II–IV° patients (*n* = 6). **d** Frequencies of CD4^+^ CD25^high^ Foxp3^+^ Tregs in PB and BM from healthy controls (*n* = 5), non-GVHD patients (*n* = 5) and aGVHD patients (*n* = 5). **e** Reversed ratios of the frequencies of Tregs in BM and PB in acute GVHD patients (*n* = 4) compared with those in healthy donors (*n* = 4) and non-GVHD patients (*n* = 4). **f** The percentages of RTEs in each CD4^+^ subset in BM from aGVHD patients (*n* = 3) and healthy donors (*n* = 7) are shown. **g** The numbers of CXCR4^+^ cells are shown in PB from patients with (*n* = 8) or without aGVHD (*n* = 18). **h**, **i** Correlation and linear regression of the Treg proportions compared with the numbers of blood platelets and megakaryocytes in bone marrow smears from patients with (*n* = 7) or without (*n* = 6) aGVHD. Each dot represents an independent core, with a two-tailed Pearson’s correlation statistical analysis including six patients in each group. **j** The numbers of CD34^+^ cells are shown in BM from patients with or without aGVHD (*n* = 6). *P < 0.05; **P < 0.001

Previous studies have verified the progressive loss of Tregs in the PB of patients with aGVHD, which is initiated by proinflammatory donor T cells. The BM is also acknowledged as a target of attack for donor T cells in aGVHD; subsequent damage to the hematopoietic stem cell niche contributes to delayed hematopoietic reconstitution.^22^ However, studies of the changes in Tregs in the BM of aGVHD patients have not produced clear results. To examine the abnormal frequencies of Tregs in aGVHD patients, we first measured the percentage of Tregs in CD4^+^ T-cell populations in both BM and PB samples from patients with or without aGVHD and in healthy donors. All aGVHD patients showed a significantly decreased frequency of Tregs in BM (Fig. 1a, b) compared with that in healthy controls (*P* < 0.0001) or non-GVHD patients (*P* = 0.006). Furthermore, the frequency of Tregs was closely related to the severity of aGVHD (*P* = 0.001) (Fig. 1c). Interestingly, BM samples from aGVHD patients had lower percentages of Tregs than PB samples (*P* = 0.021, Fig. 1d). In contrast, BM samples from the control and non-GVHD groups had higher proportions of Tregs compared with the PB samples; however, this difference was not significant (Fig. 1d). There were significantly higher numbers of Tregs in BM and PB from healthy controls and non-GVHD patients than in those from aGVHD patients. In addition, the proportions of Tregs in BM from aGVHD patients were more greatly reduced than those in the PB samples (Fig. 1e).

T cells are reconstituted via the thymic-dependent generation of T cells from donor hematopoietic progenitor cells.^23^ To examine the reconstitution of Tregs, the percentage of recent thymic emigrant cells (RTEs), defined by the coexpression of CD45RA and CD31 in patients and healthy control samples in different groups, was assessed. CD45RA^+^CD31^+^ naive CD4^+^ T cells have characteristics that apply to RTEs, similarly to thymocytes that express CD31^24^ and have a high TCR excision circle content. Thus, the assessment of CD45RA^+^CD31^+^ naive CD4^+^ T cells could be used to evaluate human thymic function^25^. After allo-HSCT, the frequency of RTEs among Tregs was significantly reduced in the aGVHD group compared with that in healthy controls (*P* = 0.030; Fig. 1f). These results indicated that Treg reconstitution in BM was impaired by the thymic generation of Tregs.

Previous data have demonstrated that Tregs are recruited by chemokines secreted in BM, such as CXCL12, which binds to its cognate receptor CXCR4 on Tregs.^26^ We hypothesized that Tregs in patients with aGVHD might have an impaired capability to home to BM in response to CXCL12/CXCR4 signaling. PB Tregs displayed significantly lower frequencies of CXCR4-positive cells in aGVHD patients compared with that in the non-GVHD group (*P* = 0.029; Fig. 1g). CXCR4 expression in Treg cells is lower in memory cells in comparison with that in naive counterpart cells.^27^ When we investigated CXCR4-positive Treg cells, we found a significantly increased proportion of CD45RA^+^ naive Treg cells compared with that of CXCR4-negative Treg cells in the same samples; most CXCR4-negative Treg cells were CD45RO^+^ memory Treg cells (Supplementary Fig. 1A). Evidence suggests that Treg cells are active in aGVHD patients and reduce their CXCR4 expression in the periphery. These results may partly explain the more serious impairment of BM Tregs compared to that of PB Tregs in aGVHD patients.

We also assessed the correlation between BM Tregs and hematopoietic reconstitution. The results indicated that the proportion of Tregs was correlated with the number of blood platelets (*r* = 0.320, *p* = 0.040) and megakaryocytes in the BM smear (*r* = 0.370, *p* = 0.030) (Fig. 1h, i). However, the proportion of Treg cells was not obviously correlated with the number of neutrophils (*r* = 0.16, *p* = 0.6) or reticulocytes (*r* = 0.03, *p* = 0.9) (Supplementary Fig. 1B and 1C). We tested CD34^+^ cells in BM samples and found lower levels in aGVHD patients compared with those in the non-GVHD group (Fig. 1j). These results provide further evidence regarding the impact of BM Treg deficiency on hematopoietic stem cell engraftment in humans.

### Impaired function and stability of Tregs from aGVHD patients

**Fig. 2:**
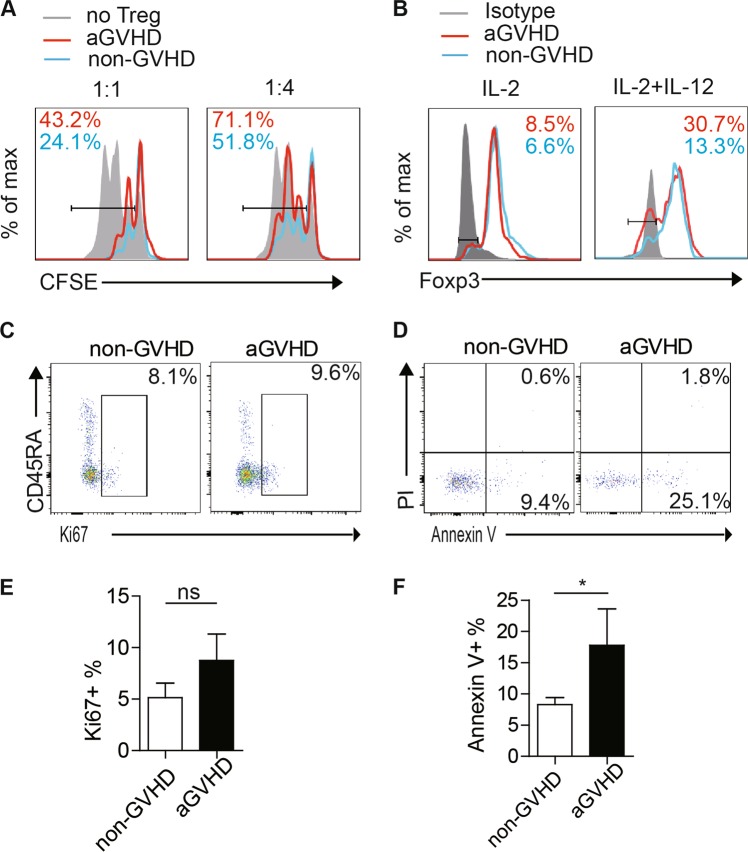
Impaired function and stability of Tregs from aGVHD patients. **a** Suppression of the proliferation of CFSE-labeled T cells (responding cells, Tresp) by different ratios of CD4^+^ CD25^high^CD127^low^ Tregs from patients with or without aGVHD. Tresp cell division was determined by CFSE dilution assays with the indicated ratios of Tregs and Tresps. **b** Foxp3 expression in Tregs from aGVHD patients and non-GVHD patients cocultured with DCs supplemented with the indicated cytokines. **c**, **e** Ki67 staining of Tregs from GVHD and non-GVHD patients (*n* = 4). **d**, **f** Annexin V and PI staining of Tregs from GVHD (*n* = 4) and non-GVHD patients (*n* = 13). *P < 0.05; **P < 0.001

Tregs have the important function of suppressing effector T cells and maintaining Foxp3 expression.^28^ Some extreme conditions may cause the loss of Tregs or change their lineage identity, leading to immune imbalance and diseases. The Treg cells obtained from aGVHD and non-GVHD patients were isolated before the starting of specific treatments, such as anti-CD25 or anti-CD52 monoclonal antibodies and mesenchymal stem cells (MSCs), except for the routinely prophylactic application of cyclosporine or tacrolimus after transplantation. After coculture with responder T cells (Tresp) at different ratios, the BM-derived Tregs from aGVHD patients could not efficiently inhibit Tresp proliferation, unlike those from the non-GVHD group (Fig. 2a). Treg stability was detected by using a dendritic cell-Treg coculture system, which could imitate physiological states.^29^ Tregs in the aGVHD group showed no obvious loss of stability after coculture with dendritic cells and IL-2 for 3 days (Fig. 2b); however, after adding IL-12, the significant loss of Foxp3 expression was observed (Fig. 2b). Previous studies have also shown abnormally increased levels of IL-12, a potent STAT4 activator, in sera from aGVHD patients.^30,31^ These results implied that Treg instability in response to IL-12 might contribute to the loss of Tregs in aGVHD patients.

We also tried to explore whether Tregs from aGVHD and non-GVHD groups differed in terms of the proliferation and apoptosis pathways. The proliferation of Tregs from the aGVHD group was barely altered compared with that in Tregs from the non-GVHD group (Fig. 2c, e). However, Tregs from the aGVHD group had a higher apoptosis rate, as assessed by Annexin V and PI staining, than those from the non-GVHD group (Fig. 2d, f).

### Transcription signatures of Tregs in patients with or without aGVHD

**Fig. 3:**
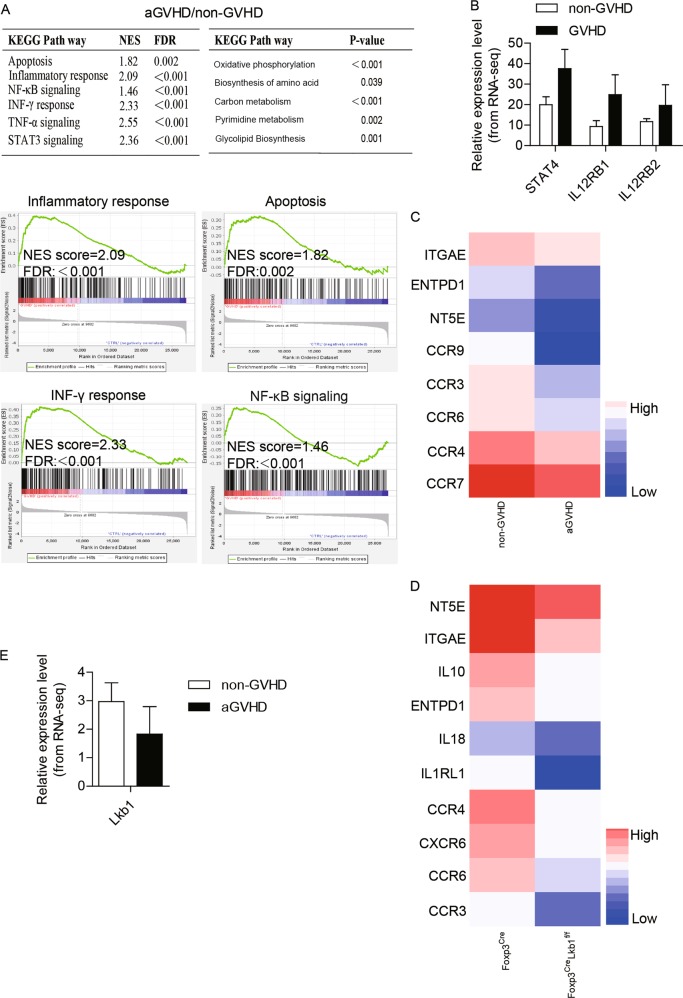
Transcriptional signature of Tregs in patients with or without aGVHD. **a** Gene pathways that were differentially expressed in Tregs from non-GVHD patients and aGVHD patients according to Gene Set Enrichment analysis. Gene sets were considered statistically significant at an FDR *P*-value < 0.05. **b** Expression profile of STAT4, IL12RB1 and IL12RB2 in Tregs according to RNA sequencing. **c** Treg function-related genes differentially expressed between CD4^+^ CD25^high^CD127^low^ Tregs from patients with or without aGVHD as determined by transcriptional profiling and shown in groups based on their function. The fold difference represents the fold change in the gene expression level in Tregs from patients with aGVHD compared with that in Tregs from non-GVHD patients. **d** Treg function-related gene expression in CD4^+^YFP^+^ Tregs from Foxp3^Cre/+^ and Foxp3^Cre/+^Lkb1^f/f^ mice as determined by transcriptional profiling. **e** Expression profile of Lkb1 in Tregs according to RNA sequencing

To explore the possible mechanisms leading to the multiple defects in Tregs from aGVHD patients, the transcription profiles of Tregs from patients with or without aGVHD were measured and analyzed. Remarkably, aGVHD led to extensive transcriptional changes in Tregs; among the 27550 gene transcripts, 1128 with a ≥ 2 or ≤ −2-fold change were detected. Pathway enrichment analysis was performed using the Kyoto Encyclopedia of Genes and Genomes pathway database and gene-set enrichment analysis.^32,33^ In Tregs from aGVHD patients, pathways involved in the inflammatory response, apoptosis, INF-γ response, and TNFα signaling (Fig. 3a) were identified using gene-set enrichment analysis. Furthermore, molecules related to the STAT3 signaling pathway were activated in Tregs from aGVHD patients (Fig. 3a). This is consistent with previous reports that STAT3 limited the number of Tregs.^12^ The results also indicated that the downregulated genes in Tregs from patients with aGVHD were enriched in metabolism-related pathways, especially those involved in oxidative phosphorylation, nucleotide metabolism, amino acid synthesis and metabolism and glycolipid biosynthesis (Fig. 3a), which was similar to the results in Foxp3^Cre^Lkb1^f/f^ mice.^21^ Interestingly, the STAT4, IL12RB1, and IL12RB2 mRNA levels were elevated in Tregs from aGVHD patients (Fig. 3b) and were associated with NF-κB signaling pathway hyperactivation in the aGVHD group (Fig. 3a). The NF-κB signaling pathway plays an important role in the development and functional divergence of different helper T-cell subsets and Tregs.^34^

To complement this approach, we used ingenuity pathway analysis to illustrate the differences in the activation of gene pathways in the various groups. The disease- and physical function-associated pathways that were highly activated in Tregs from patients with aGVHD included the inflammatory response, immune cell response, cell-to-cell signaling, and cell death. Strikingly, Tregs isolated from aGVHD patients showed the downregulation of molecules directing cells toward aGVHD target organs (liver, lungs, and intestine), such as CCR3, CCR6, CCR7, CCR9, and CCR4. In addition, molecules associated with the function of Tregs, including ENTPD1, ITGAE, and NT5E, were also downregulated in Tregs from aGVHD patients (Fig. 3c).

### Downregulation of Lkb1 in Tregs in aGVHD

Many molecules regulate the function and stability of Tregs, and of these, Lkb1 has recently been demonstrated to play an important role in maintaining the stability of Tregs based on conditional knockout mice models used in our previous study.^19^ The phenotype of the autoimmune disease of Foxp3^Cre^Lkb1^f/f^ mice is very similar to that of aGVHD mice. The molecules associated with the suppressive function of Tregs, in agreement with their defective suppressive functions in Tregs from the aGVHD group, were similar to those identified by using microarrays in Foxp3^Cre^Lkb1^f/f^ mice (Fig. 3d). Importantly, the Lkb1 mRNA level was lower in Tregs from aGVHD patients than that in the non-GVHD counterparts according to microarray analysis (Fig. 3e). We therefore considered whether the decrease in the proportion of Tregs in aGVHD patients might be relevant to the decreased expression of Lkb1 in Tregs. Altogether, these results suggested that NF-κB was activated and might affect the function of Tregs in aGVHD, which implies a similar mechanism as that revealed by our previous results in Lkb1-deficient Tregs from a mouse model.^19^ It is possible that Lkb1 is of similar importance for Tregs in aGVHD patients.

**Fig. 4:**
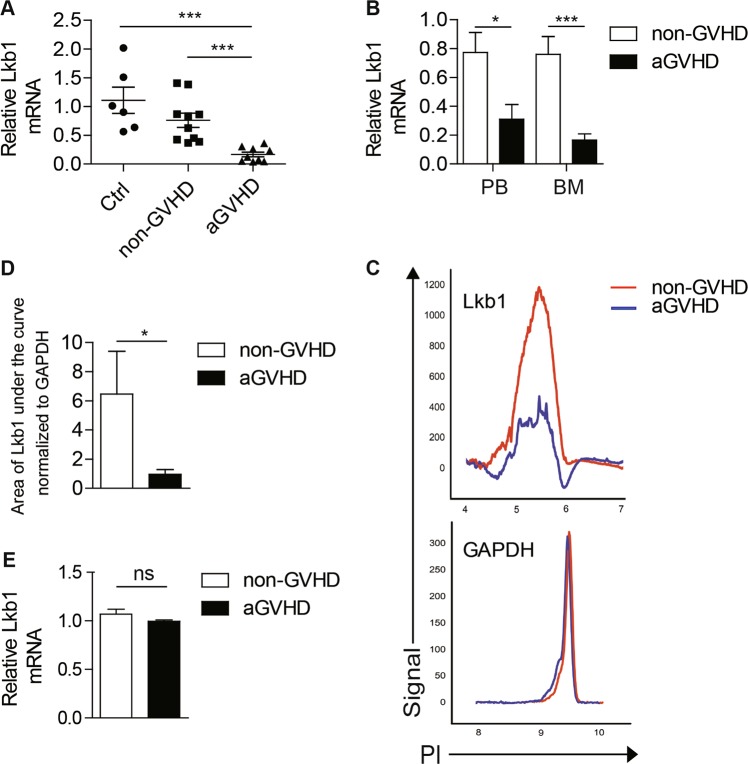
Downregulation of Lkb1 in Tregs in aGVHD patients. **a**, **b** The results of qPCR are presented as the mean values of the relative Lkb1 mRNA expression levels compared to those of the housekeeping gene GAPDH in patients with or without aGVHD and healthy donors, respectively. **c**, **d** NanoPro 1000TM system analysis. Samples were analyzed immediately after isolation without first being cultured. The peaks at pI 5.90 and pI 9.95 represent Lkb1 and GAPDH, respectively. The ratio of the peak area of Lkb1 normalized to that of GAPDH from the same sample is presented, demonstrating that non-GVHD patients (*n* = 4) had higher Lkb1 levels than aGVHD patients (*n* = 7). **e** Relative Lkb1 mRNA expression levels in samples from donors of patients with aGVHD (*n* = 3) and without GVHD (*n* = 4). *P < 0.05; **P < 0.001

To validate the transcription profile data, the expression of Lkb1 at the transcript level was measured by qPCR in Tregs from healthy donors and patients with or without aGVHD after allo-HSCT. A decreased mRNA level of Lkb1 could be observed in Tregs isolated from PB as well as BM from aGVHD patients compared with that in PB and BM from non-GVHD patients (Fig. 4a, b). Treg cells contain both naive (CD45RA^+^) and memory cells (CD45RA^−^CD45RO^+^); we sorted the CD45RA^−^CD45RO^+^ Tregs from aGVHD and non-GVHD patients and verified the levels of Lkb1 transcript expression by qPCR. The decreased expression of Lkb1 could also be observed in activated Tregs isolated from aGVHD patients compared with that in Tregs from non-GVHD patients (Supplementary Fig. 2A). The differential expression of Lkb1 at the protein level was further analyzed, and the mean protein level of Lkb1 in Tregs from aGVHD patients was found to be significantly lower (*P* < 0.001) (Fig. 4c, d) than that in non-GVHD patients.

The transcription level of Lkb1 in Tregs from donors before transplantation was further compared with that in the aGVHD and non-GVHD groups. No correlation was found between the donor Lkb1 expression level and the subsequent occurrence of aGVHD (*P* = 0.213) (Fig. 4e).

### Interference by Lkb1 affected Foxp3 expression in human Tregs

**Fig. 5:**
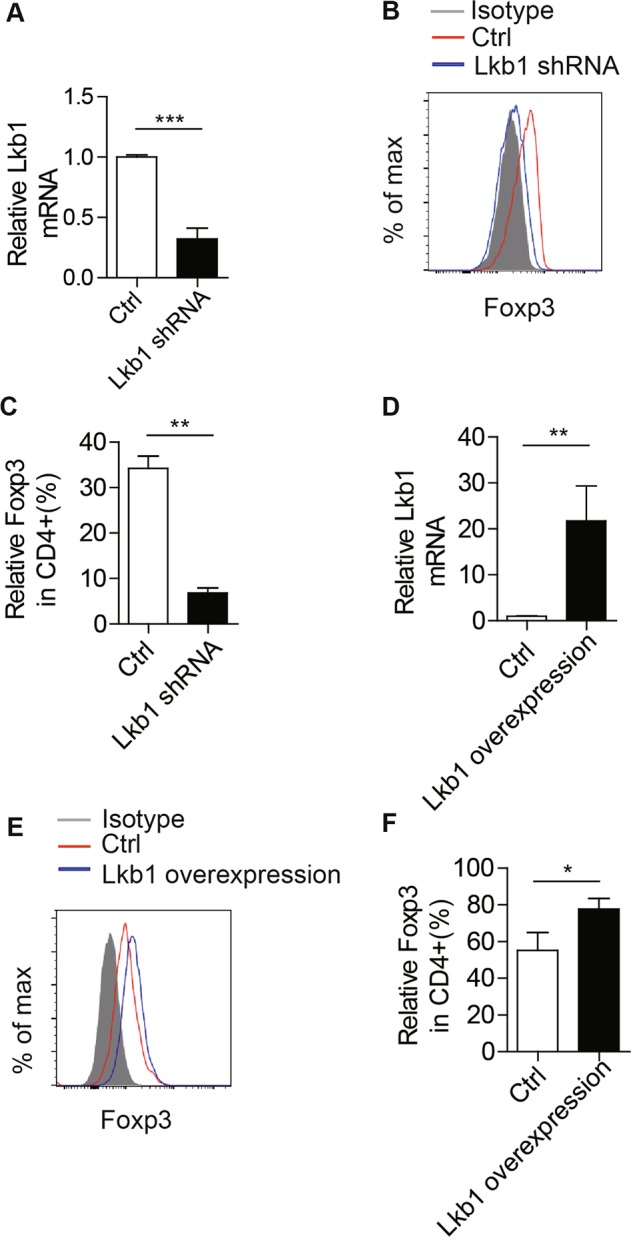
Interference by Lkb1 affected Foxp3 expression in human Tregs. **a** Determination of gene expression levels in activated Tregs treated with shRNA after 48 h by qPCR analysis. Column diagram analysis of the mRNA levels of Lkb1 after knockdown in Tregs. **b**, **c** Flow cytometric analysis of Foxp3 expression in Tregs treated with shRNA or in the control group. **d** Determination of gene expression levels in Tregs transduced with lentiviruses carrying Lkb1 complementary DNA at 48 h by qPCR analysis. Column diagram analysis of the mRNA levels of overexpressed Lkb1 in Tregs. **e**, **f** Flow cytometric analysis of Foxp3 expression in Tregs transduced with Lkb1 lentivirus or in the control group. (*n* = 3). *P < 0.05; **P < 0.001; error bars represent the s.d.; all data are representative of three independent experiments

Assuming that the function of Lkb1 was crucial for maintaining immune homeostasis during aGVHD, it was essential to address whether Foxp3 expression in human Tregs was directly regulated by Lkb1. In this experiment, specific short hairpin (sh)RNA was employed to inhibit Lkb1 in human Tregs, and qPCR analysis was performed to check its efficiency. The gene expression of glyceraldehyde 3-phosphate dehydrogenase was normalized. The Lkb1 mRNA knockdown efficiency was reduced by 60–70%, which was significantly lower than that of the control lentivirus (Fig. 5a). The knockdown of Lkb1 expression led to a significant decrease in *Foxp3* gene expression (Fig. 5b, c), which indicated that the shRNA-mediated knockdown of Lkb1 affected the stability of Tregs. Meanwhile, we overexpressed Foxp3 in Lkb1-knockdown cells for functional rescue. Compared with that in Lkb1-knockdown Treg cells, the overexpression of Foxp3 partially increased the expression of Foxp3 (Supplementary Fig. 3A). After coculture with responder T cells, Lkb1-knockdown Treg cells could not efficiently inhibit responder T cell proliferation compared with Treg cells that overexpressed Foxp3 (Supplementary Fig. 3B). To further define the precise role of Lkb1 in maintaining Tregs, a lentivirus carrying the *Lkb1 ORF* sequence was produced and transduced into Tregs from healthy donors. Lkb1 overexpression was confirmed in Tregs (Fig. 5d); the overexpression of Lkb1 partially increased the expression of Foxp3 (Fig. 5e, f).

### Lkb1 loss is correlated with CNS2 methylation in Tregs obtained from a aGVHD murine model

**Fig. 6:**
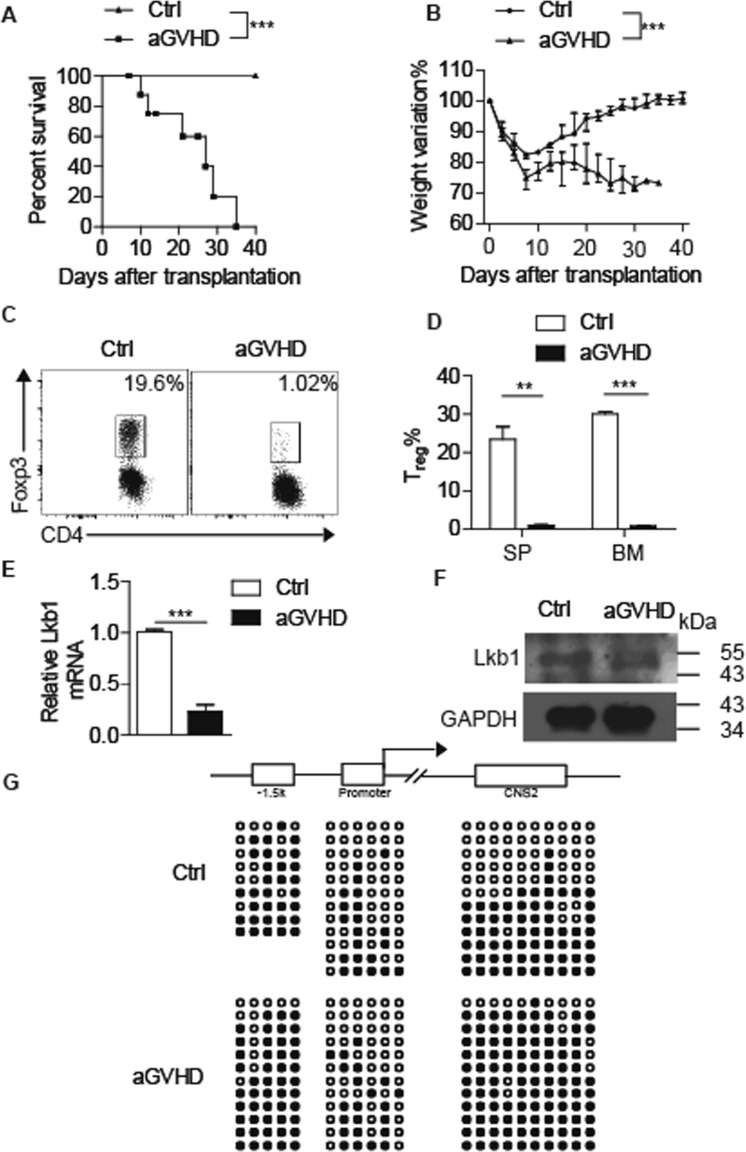
Lkb1 loss is correlated with CNS2 methylation in Tregs obtained from the aGVHD murine model. **a**, **b** Irradiated BALB/C (*n* = 9) and C57BL/6 (*n* = 9) recipients were transplanted with 45.1^+^ C57BL/6 BM and spleen cells. Overall survival and serial weight curves are depicted. **c**, **d** Foxp3^+^ Treg percentages among CD4^+^ T cells from the spleen and BM from the allogeneic aGVHD group and the syngeneic control group (*n* = 6). **e** Relative Lkb1 mRNA expression levels normalized to those of the housekeeping gene GAPDH in the aGVHD and control groups (*n* = 3). **f** Lkb1 protein in CD4^+^ CD25^high^ Tregs from spleen cells from mice with or without aGVHD by western blotting. **g** Methylation of CpG motifs in the −1.5 kb region, promoter, and CNS2 in the Foxp3 locus in Tregs from aGVHD and control mice, as determined by bisulfite sequencing. Filled circles represent methylated CpG sites, and open circles represent unmethylated CpG sites. *P < 0.05; **P < 0.001

To further explore the regulatory mechanism of Lkb1 in Tregs during aGVHD, we established a murine aGVHD model. Most mice that received allo-HSCT lost weight and died within 35 days post-BM transplantation (Fig. 6a, b). Histopathological examination showed the massive infiltration of lymphocytes and tissue damage in multiple organs, such as the liver, intestine and kidney, in the aGVHD murine model. The percentages of peripheral Tregs in the CD4^+^ T-cell populations decreased sharply in both spleen and BM from aGVHD mice (Fig. 6c, d). Lkb1 mRNA and protein levels were also reduced in the spleen and BM of aGVHD mice compared to those in syngeneic BM transplantation animals (Fig. 6e, f).

We previously reported that Lkb1 stabilized Foxp3 expression by preventing the methylation of CNS2 in the Foxp3 locus.^19^ Based on this observation, we examined the methylation of the CpG locus in various regions of Foxp3 by bisulfite sequencing in CD4^+^ CD25^+^ Tregs obtained from control mice and aGVHD mice. We found a remarkable increase (nearly 80%) in the methylation of CpG at CNS2 in aGVHD Tregs (Fig. 6g). Although the Foxp3 promoter and other possible regulatory regions are related to Treg development, the methylation of the CpG-containing promoter and −1.5 kb region in aGVHD Tregs was in line with the results observed in the control Tregs (Fig. 6g). Previous research verified the CpG demethylation of CNS2 in Foxp3 in suppressive Tregs.^35^ Our results suggest that Lkb1 loss was correlated with CNS2 demethylation in Tregs in the aGVHD murine model.

### Deletion of Lkb1 impairs Tregs and exacerbates aGVHD murine lethality

**Fig. 7:**
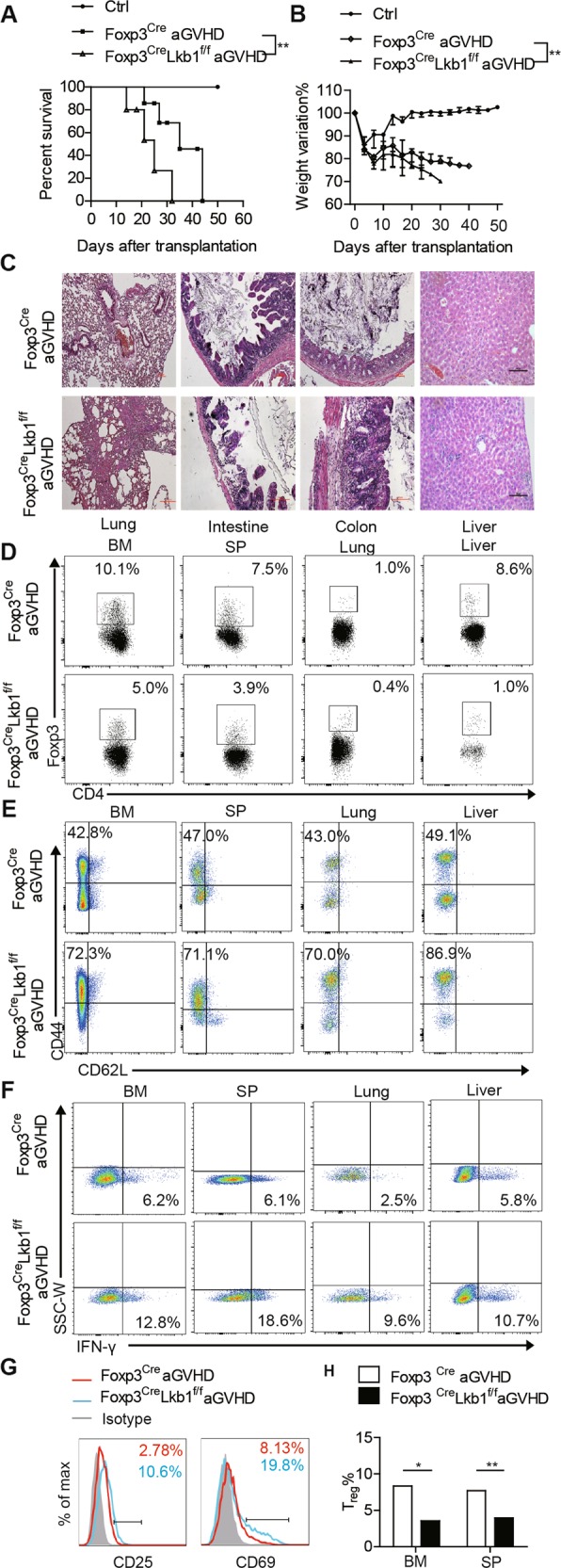
Deletion of Lkb1 impairs Tregs and exacerbates aGVHD murine lethality. **a**, **b** Lethally irradiated BALB/C mice were transplanted with 2.5 × 10^5^ CD4^+^ YFP^+^ Tregs from Foxp3^Cre^Lkb1^f/f^ and Foxp3^Cre^ mice purified by flow cytometry together with 5 × 10^6^ 45.1^+^ C57BL/6 TCD-BM cells and 1 × 10^6^ CD4^+^YFP^-^ conventional T cells from Foxp3^Cre^ mice. Overall survival and body weight curves are depicted. **c** Representative hematoxylin and eosin-stained sections of the liver, lung, intestine and colon from animals transplanted with Tregs from Foxp3^Cre^Lkb1^f/f^ and Foxp3^Cre^ mice (scale bar, 100 mm). **d**, **h** Foxp3^+^Treg cell percentages among the CD4^+^T cells in spleen, BM, lung and liver in animals transplanted with Tregs from Foxp3^Cre^Lkb1^f/f^ and Foxp3^Cre^ mice (*n* = 3). **e** Expression of CD44 and CD62L in spleen, BM, lung and liver CD4^+^ Foxp3^−^ T cells from animals transplanted with Tregs from Foxp3^Cre^Lkb1^f/f^ and Foxp3^Cre^ mice. **f** Intracellular staining of cytokines in spleen, BM, lung, and liver CD4^+^ Foxp3^−^ T cells from animals transplanted with Tregs from Foxp3^Cre^Lkb1^f/f^ and Foxp3^Cre^ mice stimulated with phorbol myristate acetate (PMA) and ionomycin for 4-5 h. **g** Expression of CD25 and CD69 in splenic CD4^+^ Foxp3^−^ T cells from mice transplanted with Tregs from Foxp3^Cre^Lkb1^f/f^ and Foxp3^Cre^ mice. All mice were analyzed 14 days after transplantation, unless otherwise specified. *P < 0.05; **P < 0.001

In our previous study,^19^ an in vitro Treg cell suppression assay showed that when responder T cells were exposed to different ratios of Treg cells from Foxp3^Cre^ and Foxp3^Cre^Lkb1^f/f^ mice, Lkb1-deficient Treg cells could not efficiently suppress the proliferation of responder T cells, indicating the impaired suppressive capacity of Lkb1-deficient Treg cells. Given that the absence of Lkb1 led to functional defects in Tregs, we assumed that Tregs highly expressing Lkb1 could alter the outcome of aGVHD after being transferred into aGVHD mice. To assess this, recipient Balb/c mice were transplanted with the same amount of a cell mixture containing BM, CD4^+^CD25^−^ conventional T cells from C57BL/6 mice and CD4^+^ Foxp3^YFP+^ Tregs from either Foxp3^Cre^Lkb1^f/f^ or Foxp3^Cre^ mice. Mice with transplanted Foxp3^Cre^Lkb1^f/f^ donor grafts showed exacerbated aGVHD mortality (Fig. 7a) and accelerated weight loss (Fig. 7b) compared with that in mice that received wild-type Foxp3^Cre^ grafts. Histological analysis revealed increased pathological damage in the liver, lung, intestine, and colon of these animals (Fig. 7c) and the infiltration of inflammatory cells. There were also strikingly increased percentages of CD44^high^CD62L^low^ cells in the spleen, BM, lung and liver and significant increases in CD4^+^ IFN-γ^+^ T cells in these organs in recipients of Foxp3^Cre^Lkb1^f/f^ marrow grafts (Fig. 7e, f). Furthermore, the Foxp3^Cre^Lkb1^f/f^ group also had higher percentages of CD4^+^ and CD8^+^ conventional T cells, which expressed the activation markers CD25 and CD69, suggesting the aggravation of the aGVHD inflammatory response (Fig. 7g). Dramatically decreased percentages of Foxp3^+^ Tregs in CD4^+^ T cell populations in the spleen, BM, lung, and liver were also detected in mice with Foxp3^Cre^Lkb1^f/f^ marrow grafts after transplantation, indicating a severe defect in immune suppression that was mediated by Tregs (Fig. 7d, h). The absolute number of Treg cells was also detected, and the results indicated that there was a lower absolute number of Treg cells in recipients of Foxp3^Cre^Lkb1^f/f^ marrow grafts compared with that in recipients of Foxp3^Cre^ marrow grafts during aGVHD onset (Supplementary Fig. 4A). Meanwhile, an in vitro Treg suppression assay showed that Treg cells from recipients of Foxp3^Cre^Lkb1^f/f^ marrow grafts could not efficiently suppress the proliferation of responder T cells (Supplementary Fig. 4B and 4C), indicating the impaired suppressive capacity of Lkb1-deficient Treg cells. These data indicated that the absence of Lkb1 caused defective Tregs and exacerbated aGVHD lethality. Recent reports have demonstrated that azacitidine (AzaC) mitigates GVHD while maintaining a robust GVL effect.^36^ The hypomethylating agent azacitidine (AzaC) was prepared in cold PBS and was injected four times subcutaneously or intraperitoneally every other day starting on day +15 after HSCT. We observed that the survival rates and weight loss of mice treated in vivo with AzaC after allogeneic HSCT were different from those of mice treated after transplantation with PBS (Supplementary Fig. 4D, E). Meanwhile, we also observed that injections of AzaC significantly improved the survival of Foxp3^Cre^Lkb1^f/f^ GVHD group mice compared with that of mice injected with the PBS control (Supplementary Fig. 4F, G).

## Discussion

Tregs play an essential role in the maintenance of immune homeostasis by inhibiting autoimmune responses, and their function depends on maintaining the stability of the lineage. aGVHD is associated with qualitative and quantitative defects in Tregs.^37^ Several reports have demonstrated a lower frequency of Tregs in PB^38,39^ or mucosa from intestinal biopsies^40^ of patients with aGVHD compared to that in patients without aGVHD.

We first confirmed the patterns of change in Tregs in BM from patients with aGVHD and the influence of Tregs on hematopoietic reconstruction after transplantation. The proportion of Tregs was more closely related to thrombocytopenia, which may be associated with mechanisms that include impaired thrombopoiesis and increased platelet consumption.^41^ In addition, the use of glucocorticoids and granulocyte colony-stimulating factor (G-CSF) in patients might be able to induce a rapid increase in neutrophils in peripheral blood, which may affect the changes in neutrophils.^42^ The current study demonstrated the decreased frequencies of Tregs in both BM and PB, and the proportions of Tregs in BM from aGVHD patients were reduced far more than those in PB. Our data suggest that the deregulation of the BM immune microenvironment, which caused decreased frequencies of Tregs, abnormal migratory potential, and defective immunosuppression, might be a reason for hematopoietic dysfunction.

We also found for the first time that patients who experienced aGVHD had significantly reduced expression of Lkb1 at the mRNA and protein levels compared with those who did not have aGVHD, and we found similar results in the aGVHD mouse model. However, we determined a negative association between the expression of Lkb1 in donor grafts and the incidence of aGVHD in patients, which might be due to the effects of the inflammatory environment. Treg cells from aGVHD patients are activated compared with those from non-GVHD patients, and the decreased expression of Lkb1 could also be observed in activated Treg cells isolated from aGVHD patients compared with those isolated from non-GVHD patients. These findings suggested that the decreased expression of Lkb1 played an important role in the occurrence and progression of human aGVHD.

A growing number of studies have found that the stability of Foxp3 expression in Tregs can be lost, and Foxp3 can exert effector-like functions in response to certain environmental triggers.^43,44^ We showed that Tregs from aGVHD patients had less capacity to suppress the proliferation of effector T cells. Furthermore, we discovered that the addition of IL-12 caused the loss of expression of Foxp3 in Tregs from patients with aGVHD but not in those from patients without aGVHD.

It was also proven that the deficiency of Lkb1 caused very severe defects in Treg stability. We therefore considered that there might be similar regulatory mechanisms at play in patients with aGVHD that caused the deficiency of Lkb1 in Tregs to result in IL-12-induced STAT4 activation, unstable Foxp3 expression, and increased IL12RB and STAT4 mRNA expression. Some investigators have demonstrated that Treg transfer at later time points posttransplantation, rather than before or at the time of transplantation, was less effective in attenuating disease severity.^45,46^ In addition, increased proliferation of Tregs after allo-HSCT is not sufficient to compensate for reduced thymic output and the increased susceptibility to apoptosis. Our results imply that the variable long-term efficacy of Treg therapy for aGVHD may be related to the loss of the stability in Tregs in these patients.

Tregs play a substantial role in the homeostasis of the immune system after transplantation. Our transcriptome studies revealed the substantially reduced expression of several chemokine receptor genes (CCR3, CCR6, CCR7, CCR9, and CCR4), which are associated with the capacity to migrate to target organs during aGVHD. Beyond these results, our findings indicated the moderate downregulation of a number of immunoregulatory genes (*ENTPD1*, *NT5E*, and *ITGAE*) in Tregs from aGVHD patients. However, the level of IL-10 was not significantly decreased in aGVHD patients compared with that in non-GVHD patients according to the microarray analysis. The role of IL-10 in GVHD pathogenesis remains ambiguous,^47,48^ and the result could also have been affected by many factors, including the heterogeneity of patient disease status and previous treatments. The metabolism of immune cells is closely related to the final functional differentiation of immune cells, and Lkb1 is required for maintaining cellular metabolism and energy homeostasis in the liver and muscle.^49,50^ The downregulated genes in Treg cells from aGVHD patients were enriched in metabolism-related pathways, indicating that Lkb1 may also regulate the metabolic pathways of Treg cells during the development of aGVHD. In Tregs from aGVHD patients, pathways involved in TNFα signaling and NF-κB signaling were both activated. TNFR2 drives the activation and enhances the function of mouse Treg cells.^51^ However, there are conflicting data on the effects of TNF-α on human Treg cells, and TNFR2 expression levels may not be involved in the pathogenesis of some disorders.^52^ Therefore, these contradictory results highlight the need for an extensive investigation of the roles of TNF-α and Lkb1 in Treg cell function in vivo in humanized preclinical models.

To determine the precise impact of Lkb1 on human Tregs, we performed a lentivirus interference/overexpression test. Our results demonstrated that Tregs have significantly decreased expression of Foxp3 associated with decreased Lkb1 expression. Furthermore, similar to what was observed in murine Tregs, Lkb1 directly promoted the expression of Foxp3 in human Tregs. Tregs can be classified as thymus-derived Treg (nTreg) cells or peripherally induced Treg (iTreg) cells, and both subtypes contribute to the inhibition of the GVH reaction. We found that most Treg cells from both the non-GVHD and aGVHD groups expressed the nTreg cell marker Helios. The expression of Helios in Treg cells from aGVHD patients (Supplementary Fig. 5B) was similar to that in Treg cells from non-GVHD patients (Supplementary Fig. 5A), which was much higher than that in CD4^+^ T cells. It is currently difficult to distinguish nTreg cells from iTreg cells. Helios is a more specific marker distinguishing nTreg cells. Therefore, we are primarily addressing the role of Lkb1 in nTreg cells in this study.

The methylation of CpG dinucleotides in the Foxp3 regions is vital for the regulation of Foxp3 expression. CNS3 is crucial for Foxp3 induction, whereas the induction of Tregs depends on the role of CNS1.^53,54^ CNS2, also named the Treg-specific demethylated region, is necessary for the gene expression and stability of Tregs.^37,55^ Therefore, the methylation status of the Foxp3 CNS2 region may indicate Treg stability in cells destined for clinical use.^56^ Our results verified the substantial methylation of CNS2 in Tregs from aGVHD mice but not in those from syngeneic transplantation mice, causing a loss of Foxp3 expression. This was consistent with previous reports that Lkb1 stabilizes Foxp3 expression by increasing the demethylated status of CNS2.^19^ Thus, our study also suggested that epigenetic modulation that maintained this stabilizing hypomethylation was also a critical mechanism to induce Foxp3 expression; hence, pharmacological therapy could be used to control aGVHD. Pharmacological DNA methyltransferase inhibitors could maintain Treg fidelity following adoptive transfer.^57^ These data partly explain the mechanism underlying current clinical interventions involving pharmacological hypomethylation for adoptive cellular immunotherapy against aGVHD.

A variety of strategies to increase the number or potency of Tregs in vivo can be used to reduce the severity of aGVHD.^58^ The treatment of humans with low doses of IL-2 has been shown to ameliorate aGVHD;^59,60^ however, IL-2 therapy has some limitations, including difficulty in predicting the most efficacious dose, off-target effects, and a short in vivo half-life.^61^ Taken together, our results demonstrated the presence of multiple defects in Tregs in human aGVHD, which were induced by Lkb1 downregulation, and highlighted the Lkb1-related pathways that might be targeted by new strategies for the future treatment of aGVHD. Genetically reprogramming Tregs to overexpress Lkb1, possibly by using clinical-grade lentiviral vectors with adoptive transfer, represents an attractive strategy to prevent or treat aGVHD. In fact, exploring how Lkb1 expression is downregulated in Tregs during the development of aGVHD is very important for targeting Lkb1-related pathways to treat aGVHD. Although many downstream pathways mediating the function of Lkb1 have been described, little is known about the upstream mechanisms regulating Lkb1 activity. Further study is necessary to define the complete mechanistic landscape of aGVHD.

## Materials and methods

### Patient characteristics

We enrolled all 46 patients treated by allo-HSCT at the Institute of Hematology, Chinese Academy of Medical Sciences, between 2017 and 2018 in this study. Patients were excluded if they had relapsed leukemia or infection at the time of sample collection. Bone marrow (BM) and peripheral blood (PB) samples were collected within 3 months after transplantation. The non-GVHD group included patients who, during the first three months, had no need for aGVHD treatment; the aGVHD group included those who needed aGVHD treatment, and samples were collected on the day of onset. We also studied 10 age-matched healthy adults as controls. The clinical characteristics of the patients are summarized in Table 1. All patients received standard immunosuppressive regimens for GVHD prophylaxis. All patients and donors gave written informed consent in accordance with the Declaration of Helsinki, and the work was approved by the Ethics Committee of the Institute of Hematology, Chinese Academy of Medical Sciences (Tianjin, China).

**Table 1:** Patients and transplant characteristics

Characteristics	
Patients	46
Gender	
Male/Female	27/19
Median age (range, year)	35 (7–63)
Diagnosis	
AML	14
ALL	12
MDS	10
AA	7
Other	3
Donor type	
MSD	20
MUD	3
MMSD	23
GVHD prophylaxis	
CsA based	19
FK506 based	27
Conditioning regimen	
BCFA	27
FC + ATG	5
TCFA	14
Acute GVHD grade	
0	26
I	5
II	3
III	6
IV	6
GVHD target organ	
Skin	13
Liver	8
Intestine	15
Oral cavity	1

*MSD* matched sibling donor, *MUD* matched unrelated donor, *MMSD* mismatched sibling donor, *CsA* cyclosporin, *FK506* tacrolimus, *BCFA* busulfan, cyclophosphamide, fludarabine, Ara-c, *FC* *+* *ATG* fludarabine, cyclophosphamide, antithymocyte globulin, and *TCFA* total body irradiation, cyclophosphamide, fludarabine, Ara-c

### Mice

C57BL/6 (H-2b), BALB/c (H-2d), CD45.1^+^, Lkb1^f/f^, and Foxp3^Cre^ mice were bred in the animal facility at the Institute of Hematology, Chinese Academy of Medical Sciences, or purchased from The Jackson Laboratory (Bar Harbor, ME, USA). Lkb1^f/f^ mice were crossed with Foxp3^Cre^ mice to generate Foxp3^Cre^Lkb1^f/f^ mice. C57BL/6 (H-2b), BALB/c (H-2d), and CD45.1^+^ mice were used between the ages of 8 and 12 weeks, and sex-matched combinations were used for transplant experiments. Lkb1^f/f^Foxp3^Cre^ mice were used at 3–4 weeks, with age- and sex-matched Foxp3^Cre^ mice serving as controls. All animals were raised under specific pathogen-free conditions, and all experimental protocols were performed with the approval of the Institutional Animal Care and Use Committee of the Institute of Hematology.

### Acute GVHD and transplantation models

To induce a major histocompatibility complex-mismatched aGVHD model, we transplanted CD45.1^+^ B6 mice-derived BM along with splenic T-cells into BALB/c mice. The host allogeneic (BALB/c) or syngeneic (C57BL/6) mice at 8–12 weeks were irradiated with 800 cGy split into two doses on day 0. The recipients were then intravenously injected with 1 × 10^7^ T cell-depleted BM cells together with 1 × 10^7^ splenic cells from CD45.1^+^ C57BL/6 mice. The BM and splenic T-cell suspensions were prepared using leg bones and splenocytes, respectively. For some experiments, BALB/c mice were transplanted with 2.5 × 10^5^ CD4^+^YFP^+^ Tregs from Foxp3^Cre^Lkb1^f/f^ and Foxp3^Cre^ mice, which were purified by flow cytometry using a FACSAria III (BD Biosciences), together with 5 × 10^6^ T cell-depleted BM cells in combination with 1 × 10^6^ CD4^+^YFP^−^ conventional T cells from Foxp3^Cre^ mice. Mouse survival, body weight scores and clinical GVHD scores were assessed as described elsewhere.^62^

### Cell sorting and flow cytometry

BM and PB mononuclear cells from patients with or without aGVHD were separated using Ficoll-Hypaque density gradient centrifugation. For in vitro functional assays, specific cell populations were sorted using a FACSAria III (BD Biosciences) cell sorter into different CD4^+^ T cell subsets that were defined by antigen expression patterns as follows: CD4^+^CD25^+^CD127^−^ for Tregs and CD4^+^CD25^−^ for responder T cells (Tresp).

The indicated T cell populations from the mouse model were sorted from purified CD4^+^ T cells with the FACSAria III; the sorted populations were higher than 98% purity unless otherwise specified. Cell surface markers were stained in 1 × phosphate-buffered saline containing 2% fetal bovine serum with the indicated antibodies, which were purchased from Biolegend and eBioscience. For intracellular cytokine staining, cells were stimulated for 4–5 h with phorbol myristate acetate (50 ng/ml) and ionomycin (500 ng/ml). Flow cytometry data were acquired using a FACSCanto II (BD Biosciences) and analyzed using FlowJo software (FlowJo LLC, Ashland, Oregon).

### In vitro Treg suppression assay

For the suppression assay, freshly purified CD4 ^+^CD25^−^ T cells from the same healthy donors were labeled with CellTrace carboxyfluorescein diacetate succinimidyl ester (Life Technologies, Carlsbad, CA, USA) and used as Tresps. The Tresps (1 × 10^4^) were cultured in 96-well round-bottom plates with soluble anti-CD3 (1 μg/mL) and anti-CD28 (1 μg/mL) in the absence or presence of various numbers of Tregs from patients with or without aGVHD for 72 h. The carboxyfluorescein diacetate succinimidyl ester profiles and Foxp3 expression were examined by flow cytometry after culturing.

### Histopathological examination

Representative mice were sacrificed on the specified days, and the spleen, liver, lung, small intestine, colon, and kidney were formalin-fixed and paraffin-embedded; 5-μm-thick sections were stained with hematoxylin and eosin for histological examination. aGVHD lesions in each sample were examined at ×10 or ×20 magnification.

### Genomic DNA isolation, bisulfite treatment, and methylation profiling

Genomic DNA was purified from Tregs using the Wizard Genomic DNA Purification Kit (Promega, Madison, WI, USA) and bisulfite-transformed with the EZ DNA Methylation Direct Kit (P-1026-050, Epigentek, Farmingdale, NY, USA) according to the manufacturers’ instructions. Specific polymerase chain reaction (PCR) primers were used for the amplification of the promoter and other loci of Foxp3. The PCR products were cloned with the pGEM-T Easy Vector System (Promega Corporation) and sequenced.

### RNA sequencing and quantification of gene expression by real-time PCR

Total RNA was extracted with TRIzol (Life Technologies) and the RNeasy Mini Kit (Qiagen) from Tregs that had been purified by fluorescent-activated cell sorting from patients with or without aGVHD. The RNA was then reverse-transcribed and amplified, and sequencing libraries were generated using the NEBNext® Ultra™ RNA Library Prep Kit for Illumina® (NEB, Ipswich, MA, USA). After the extraction of total RNA, cDNA was prepared using the One Step SYBR PrimeScript RT-PCR Kit (TaKaRa Bio Inc.). Quantitative real-time PCR was performed with the GeneAmp 7500 Sequence Detection System (Applied Biosystems) using the Brilliant SYBR Green QPCR Core Reagent Kit (Stratagene).

### Western blotting

CD4^+^CD25^+^ Tregs from aGVHD and control mice were sorted and lysed. The Western blotting was carried out as previously described,^63^ with antibodies against Lkb1 (D60C5) and GAPDH (D16H11) (Cell Signaling Technology).

### NanoPro assay

Tregs were sorted and lysed in a solution containing Bicine/CHAPS Lysis Buffer, DMSO Inhibitor Mix and Aqueous Inhibitor Mix (all from Protein Simple, Santa Clara, CA, USA). The 384-well sample plate was prepared with the sample mixture solutions, including the Premix and pI Standard, anti-Lkb1 primary antibody, anti-human IgG-HRP secondary antibody, and luminol peroxide. The NanoPro 1000 (Protein Simple) was loaded and run. The chemiluminescent peaks and visually optimized tracings were analyzed using Compass 2.5.11 software.

### Lentivirus transduction

The lentiviral vector pCDH-CMV-MCS-EF1-copGFP, containing short hairpin RNAs against a nontarget control and human *STK11*, were produced in 293 T cells. Tregs from healthy donors were cultured on plates with Human T-Activator CD3/CD28 Dynabeads transduced in virus-containing media supplemented with polybrene by centrifugation for 1 h at 500 RCF. The GFP^+^ cells were then sorted for further analysis. Tregs were transduced with lentiviruses encoding human Lkb1 shRNA. Changes in Lkb1 expression were then measured using quantitative PCR (qPCR).

### Statistical analysis

Data were analyzed using Prism 5.00 (GraphPad Software, San Diego, CA, USA) to calculate the statistically significant differences in the mean values and to determine the *P* values. Survival curves were generated using the Kaplan–Meier method and compared using a log-rank test. Student’s *t* test, a paired test or two-way analysis of variance was performed, depending on the number of compared groups. *P* < 0.05 was considered statistically significant.

## Supplementary information

Supplementary Figure legends

Supplemental Figure 1

Supplemental Figure 2

Supplemental Figure 3

Supplemental Figure 4

Supplemental Figure 5
