# Supplementary material for: Loss of Lkb1 impairs Treg function and stability to aggravate graft-versus-host disease after bone marrow transplantation
Source: Cell Mol Immunol. 2019 Oct 29;17(5):483–95. doi: 10.1038/s41423-019-0312-3 (PMC7192841; doi:10.1038/s41423-019-0312-3)
Supplement: Supplementary file 1 — Supplementary Figure legends [file 41423_2019_312_MOESM1_ESM.docx]

**Supplementary Figure 1**

(A) PBMCs were stained with CD4, CD25, Foxp3, CD45RA, CD45RO and CXCR4. CD45RA or CD45RO expression in the CXCR4^+^ and CXCR4^-^ Treg cell subsets (n=6). (B) and (C) Correlation and linear regression of the Treg proportions compared to the numbers of neutrophils and reticulocytes from patients with (n=7) or without (n=6) aGVHD.

**Supplementary Figure 2**

(A) The qPCR results are presented as the mean values of the relative Lkb1 mRNA expression levels compared to those of the housekeeping gene GAPDH in CD45RA^-^CD45RO^+^ activated Tregs in patients with (n=5) or without (n=6) aGVHD.

**Supplementary Figure 3**

(A) Flow cytometric analysis of Foxp3 expression in Lkb1-knockdown Tregs or Lkb1-knockdown Tregs that overexpressed Foxp3. (B) Suppression of the proliferation of CFSE-labeled T cells (responding cells, Tresp) by Lkb1-knockdown Tregs or Lkb1-knockdown Tregs that overexpressed Foxp3.

**Supplementary Figure 4**

(A) The absolute number of Treg cells in the spleen was determined in recipients of Foxp3^Cre^Lkb1^f/f^ marrow grafts compared with that in recipients of Foxp3Cre marrow grafts during aGVHD onset. (n=3). (B) and (C) Suppression of the proliferation of CFSE-labeled T cells (responding cells, Tresp) by Tregs in recipients of Foxp3^Cre^Lkb1^f/f^ marrow grafts compared with that in recipients of Foxp3Cre marrow grafts (n=3). (D) and (E) Irradiated BALB/C recipient mice were transplanted with 45.1^+^ C57BL/6 BM and spleen cells. Treatment with AzaC (n=5)/PBS (n=5) (every other day; 4 doses) started on day 15 after HSCT. Overall survival and body weight curves are depicted. (F) and (G) Lethally irradiated BALB/C mice were transplanted with 2.5×10^5^ CD4^+^ YFP^+^ Tregs from Foxp3^Cre^Lkb1^f/f^ (KO) mice purified by flow cytometry, together with 5×10^6^ 45.1^+^ C57BL/6 TCD-BM cells and 1×10^6^ CD4^+^ YFP^-^ conventional T cells obtained from Foxp3^Cre^ mice. Following treatment with AzaC (n=3)/PBS (n=3) (every other day, 4 doses) starting on day 15 after HSCT, the overall survival and body weight curves were obtained.

**Supplementary Figure 5**

PBMCs were stained with CD4, CD25, Foxp3 and Helios. Helios expression by Tregs and the CD4^+^ T cell subsets was shown in non-GVHD patients (A) and aGVHD patients (B). All data are representative of at least three independent experiments.
