# Supplementary figures and images for: Loss of Lkb1 impairs Treg function and stability to aggravate graft-versus-host disease after bone marrow transplantation
Source: Cell Mol Immunol. 2019 Oct 29;17(5):483–95. doi: 10.1038/s41423-019-0312-3 (PMC7192841; doi:10.1038/s41423-019-0312-3)

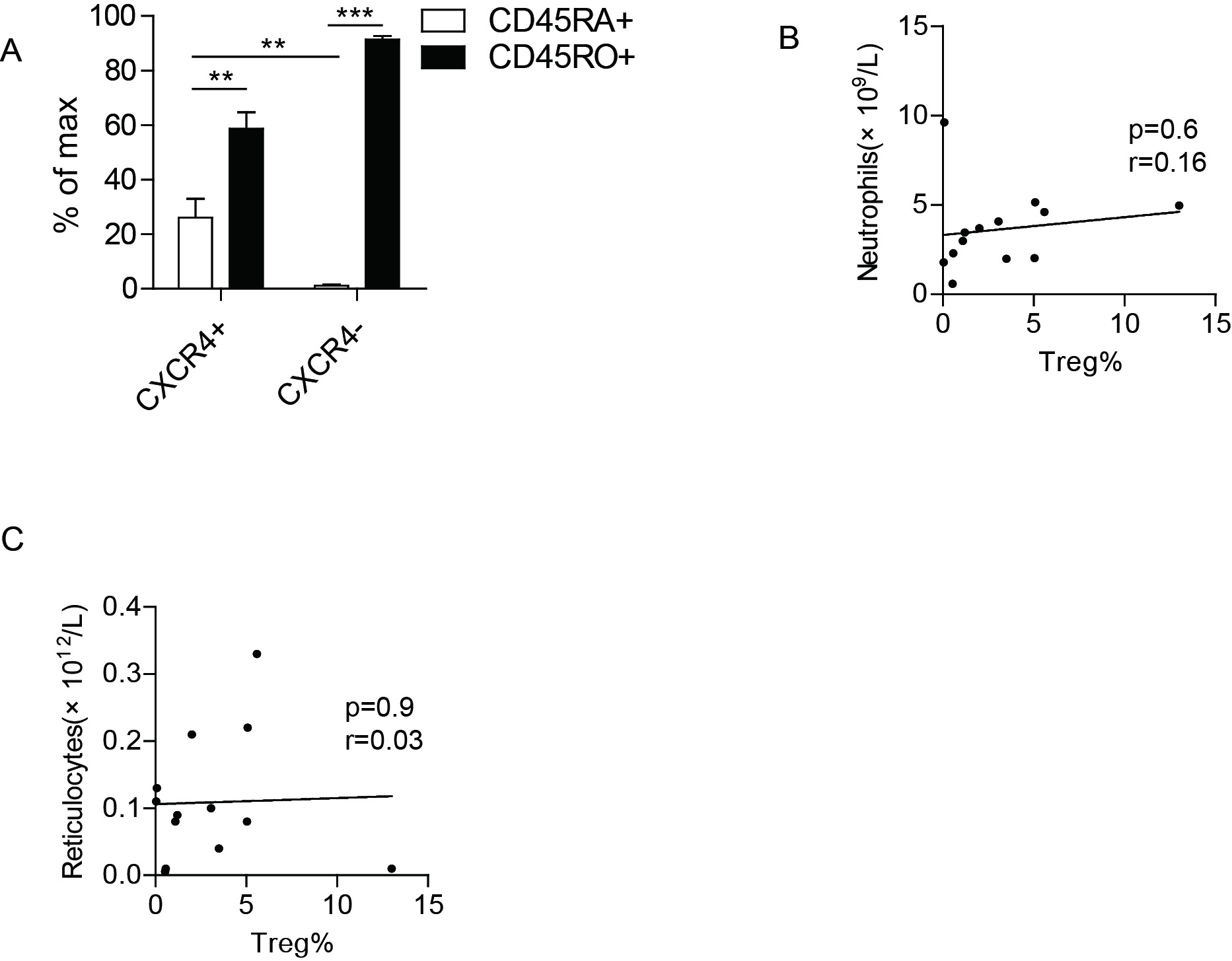

Supplement: Supplementary file 2 — Supplemental Figure 1 [file 41423_2019_312_MOESM2_ESM.tif]

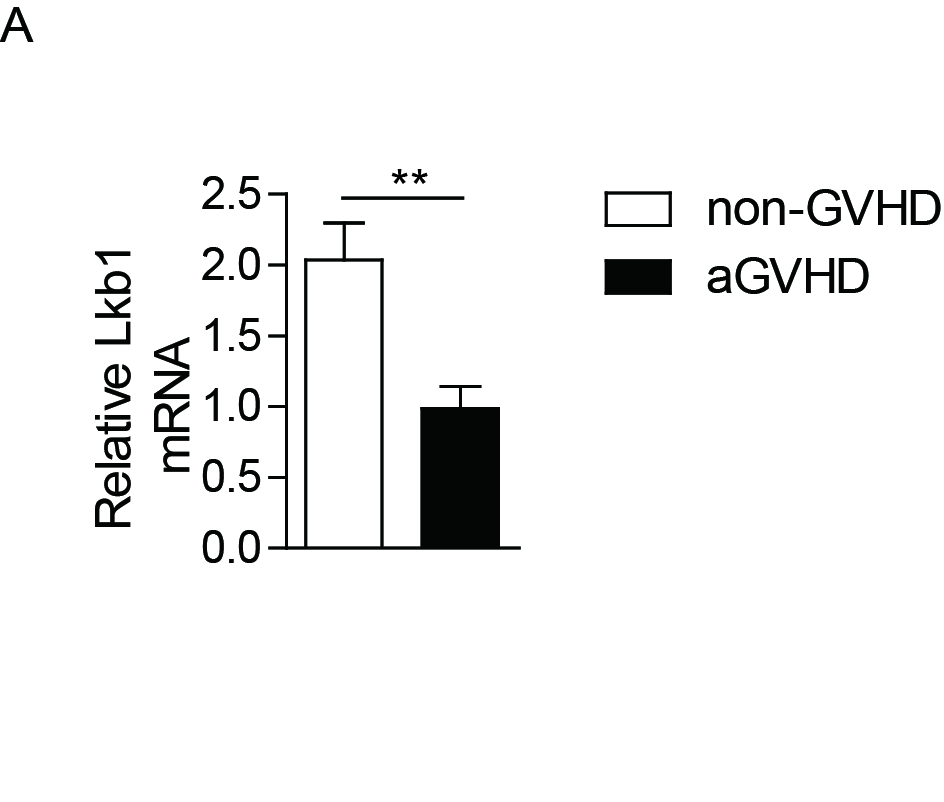

Supplement: Supplementary file 3 — Supplemental Figure 2 [file 41423_2019_312_MOESM3_ESM.tif]

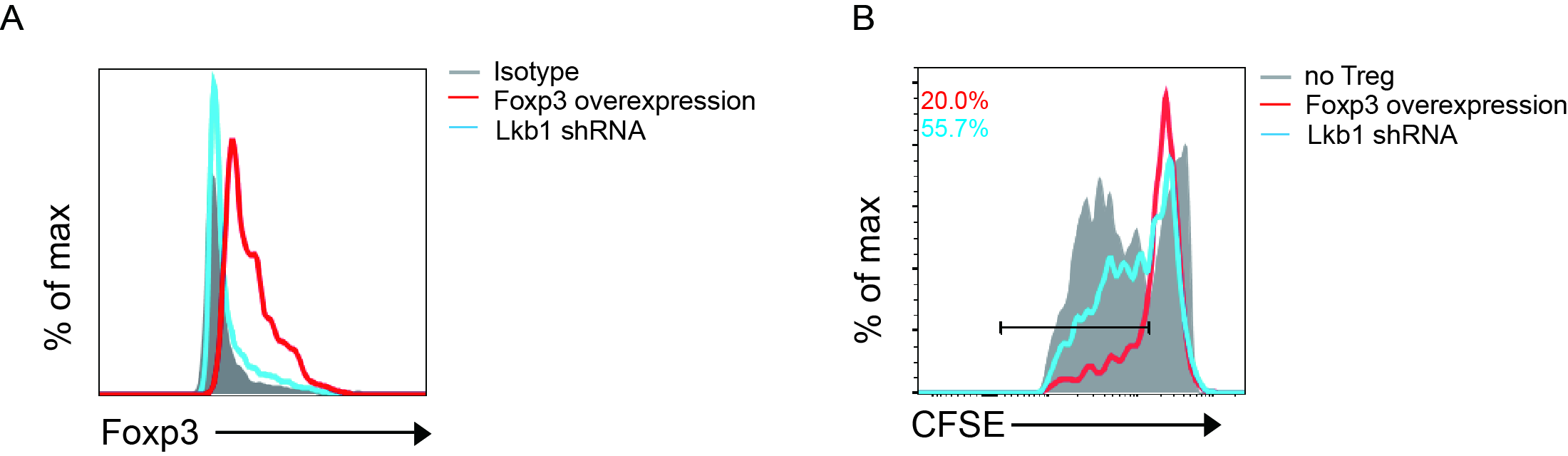

Supplement: Supplementary file 4 — Supplemental Figure 3 [file 41423_2019_312_MOESM4_ESM.tif]

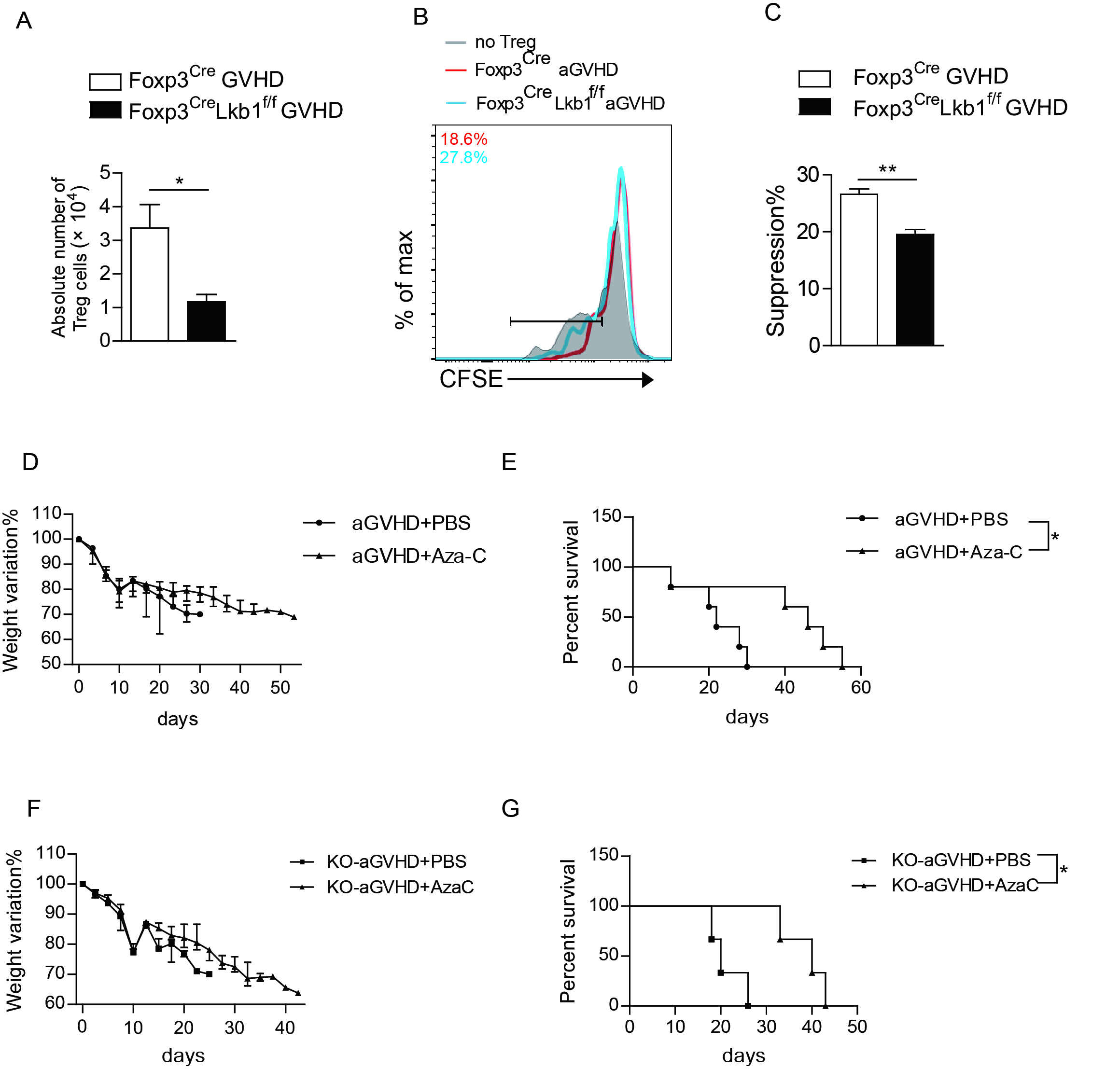

Supplement: Supplementary file 5 — Supplemental Figure 4 [file 41423_2019_312_MOESM5_ESM.tif]

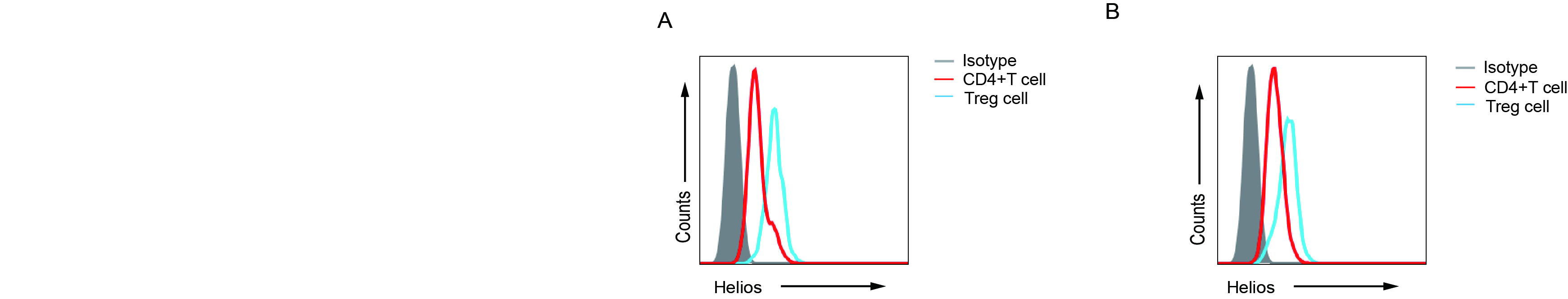

Supplement: Supplementary file 6 — Supplemental Figure 5 [file 41423_2019_312_MOESM6_ESM.tif]
